# Supplementary material for: Bedaquiline inhibits the yeast and human mitochondrial ATP synthases
Source: Commun Biol. 2020 Aug 19;3:452. doi: 10.1038/s42003-020-01173-z (PMC7438494; doi:10.1038/s42003-020-01173-z)
Supplement: Supplementary file 1 — Supplementary Information [file 42003_2020_1173_MOESM1_ESM.pdf]

Supplementary Information

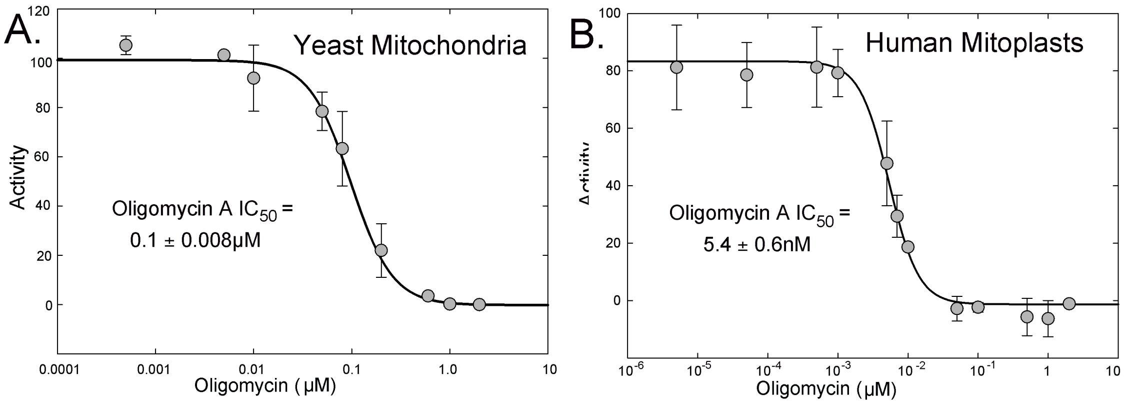

Supplement Figure 1: Inhibition of the ATP synthase activity with oligomycin. A. Inhibition of ATP synthesis of yeast mitochondria. B. Inhibition of ATP synthesis of human mitoplasts isolated from HEK293 cells. (n=3) This data was obtained using 3 independent preparations of mitochondria or mitoplasts, which were flash frozen and stored in liquid nitrogen.

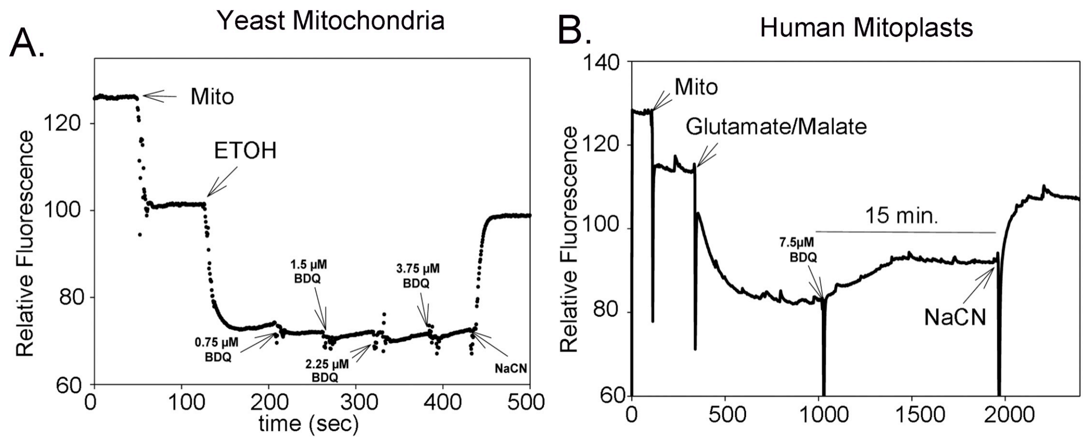

Supplement Figure 2. Affect of bedaquiline on mitochondrial membrane potential. Membrane potential was measured by fluorescence quenching of Rhodamine 123 (excitation at 488 nm and emission at 525 nm. A**) Yeast mitochondria. B) Human mitoplasts**. Rhodamine 123 (1.0 µM), mitochondria/mitoplasts (mito, 150 µg), ethanol (68 mM, EtOH), glutamate/malate (5.0mM/2.5mM), BDQ (as indicated), NaCN (0.5 mM).

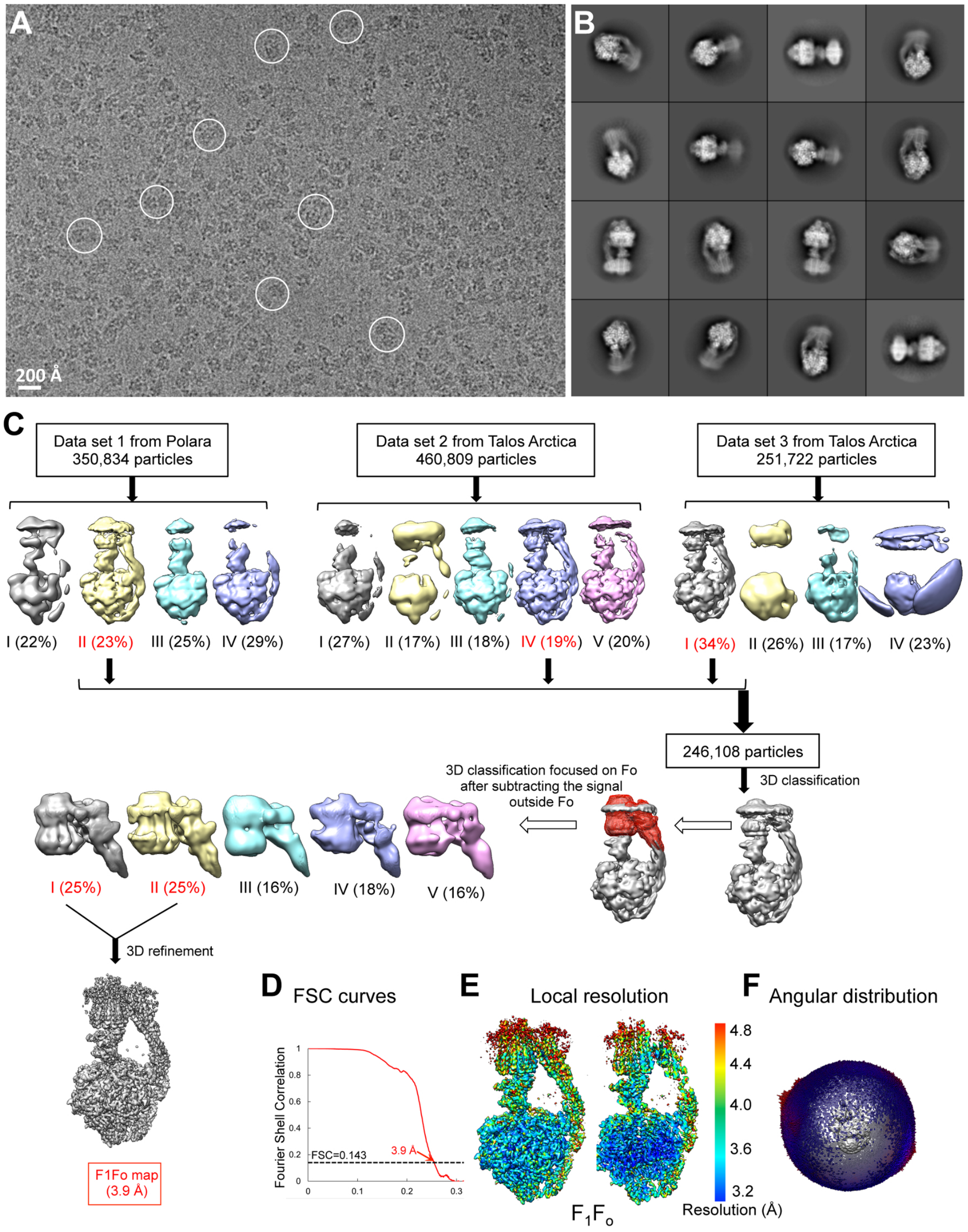

Supplement Figure 3. Single-particle cryo-EM analysis of nanodisc-embedded ATP synthase bound with bedaquiline. A, Representative cryo-EM image with several particles marked by circles. B, 2D averages of cryo-EM particle images. The box dimension is 394 Å. C, Image processing flowchart. The final F_1F_o and F_o maps with their overall resolutions are indicated in red boxes. D, Gold-standard FSC curve between two half maps that were calculated from two half data sets. E, Surface (left) and cross-sectional (right) views of the cryo-EM maps filtered to their estimated overall resolution and colored according to local resolution. F, Euler angle distribution of the cryo-EM particles for calculating the final 3D reconstruction.

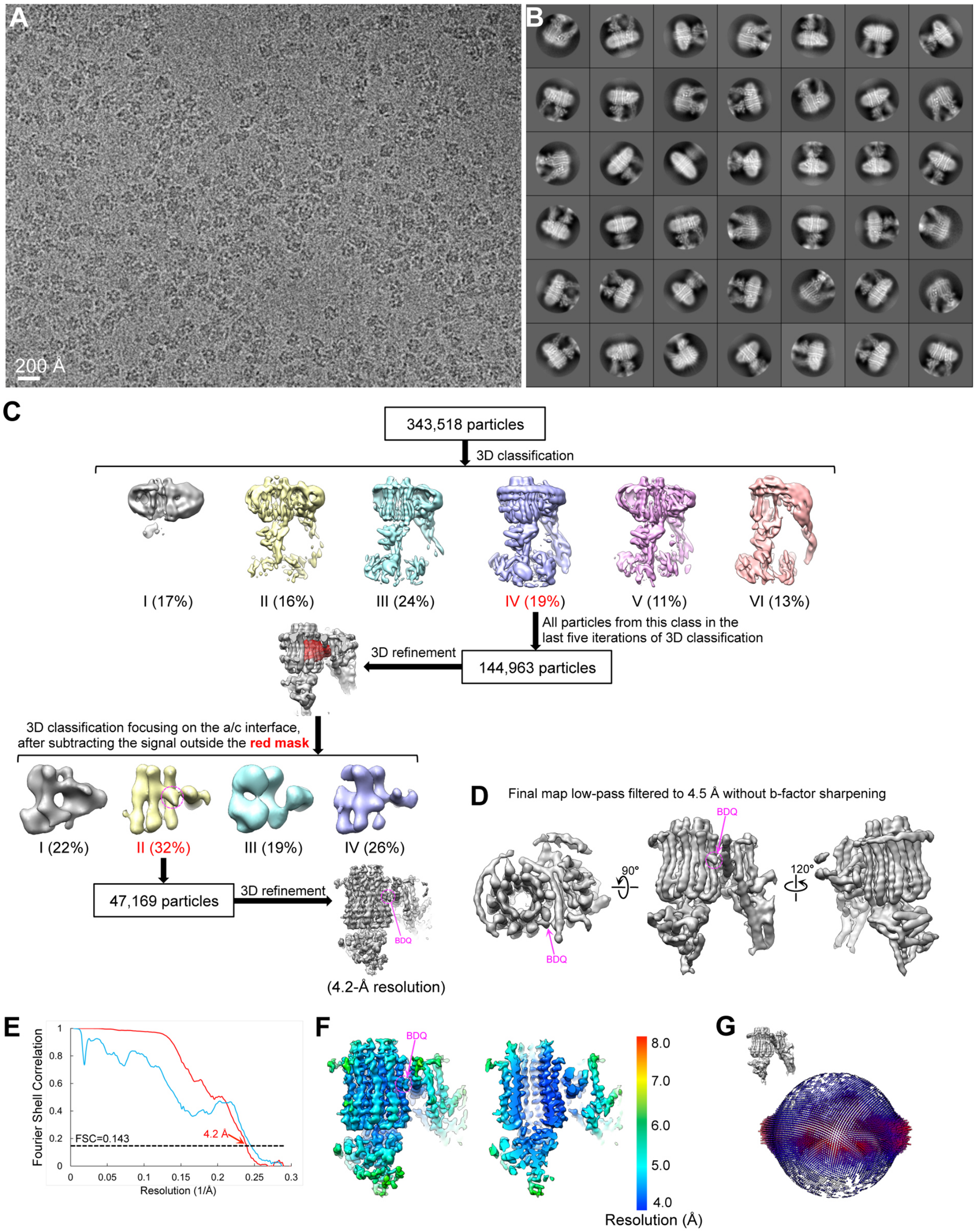

Supplement Figure 4. Single-particle cryo-EM analysis of nanodisc-embedded ATP synthase bound with bedaquiline. A, Representative cryo-EM image. B, 2D class averages of cryo-EM particle images centered on Fo. The box dimension is 238 Å. C, Image processing flowchart. D, Different views of the final cryo-EM map low-pass filtered to 4.5 Å without b-factor sharpening. The density for bedaquiline (BDQ) is indicated. E, Gold-standard FSC curves between two half maps that were calculated from two half data sets (in red), and between the summed map and the final atomic model (in blue). F, Surface (left) and cross-sectional (right) views of the final cryo-EM map, colored according to local resolution. G, Euler angle distribution of the cryo-EM particles for calculating the final 3D reconstruction.

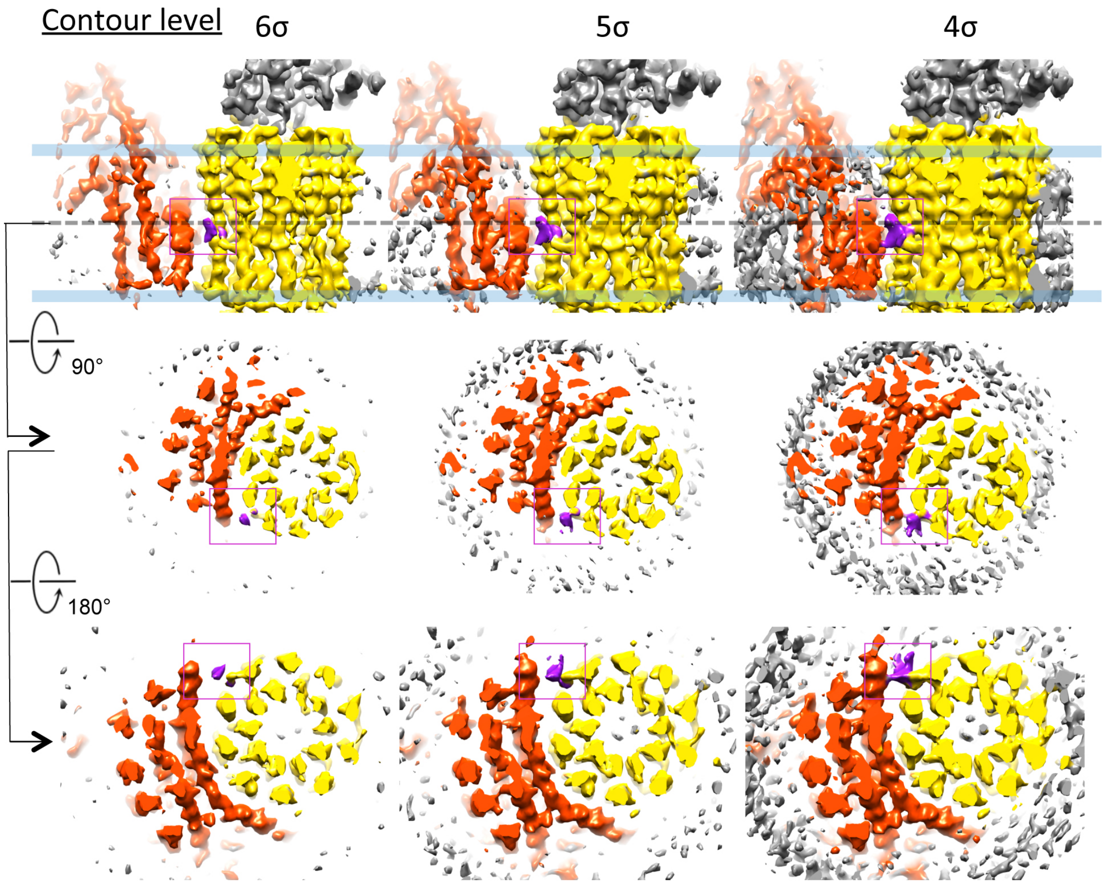

Supplement Figure 5. Cryo-EM map of F_o bound with BDQ at different contour level: 6σ, 5σ, 4σ. The density of c-ring is colored in gold and the rest subunits of Fo are colored in red, the density of central stalk and the lipids is in grey, density corresponding to bound BDQ is highlighted and colored in purple.

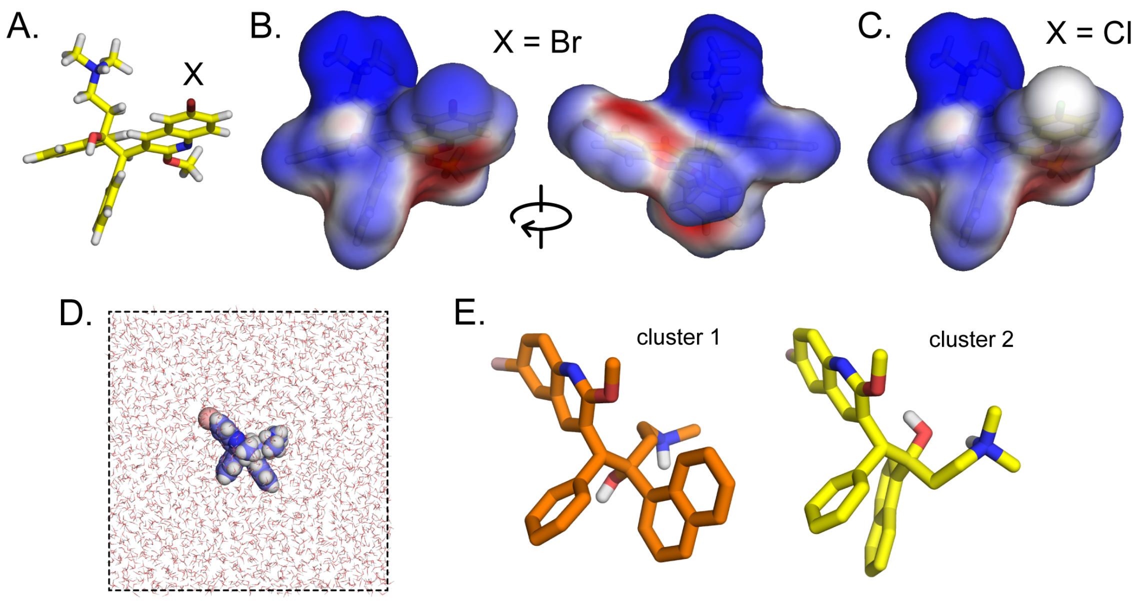

Supplement Figure 6. Development of CHARMM36-compatible parameters for bedaquiline. (A-C) Quantum-mechanical calculations of the electronic structure of bedaquiline. (A) Atomic structure of bedaquiline (carbon, yellow; nitrogen, blue; oxygen, red; hydrogen, white). The atom denoted with X is bromine. (B) Electrostatic potential generated by bedaquiline on a virtual surface that is 0.5 Å beyond the solvent-accessible surface. Two different views are shown. The electrostatic potential, colored from red to blue (-40 k_BT/e to 40 k_BT/e), reflects atomic charges derived quantum-mechanically, based on optimized geometries (Methods). The calculations correspond to the protonated form of the amine group, i.e. the total charge of the inhibitor is +1e. (C) Same as (B) upon substitution of bromine by chlorine. The electrostatic potential calculations in (B) and (C) used the APBS plug-in of Pymol. The atomic radii *r* used in these calculations, in Å, were *r* (H) = 1.1, *r* (C) = 1.75, *r* (O) = *r* (N) = 1.6, *r* (Cl) = 1.75, *r* (Br) = 1.85, and the dielectric constant was set to 1. (D-E) Molecular dynamics simulation of BDQ in water. (D) Simulation system, including one BDQ molecule (spheres) and 2,962 water molecules, enclosed in a periodic box of 45 x 45 x 45 Å. The total number of atoms is approx. 9,000. (E) The two most populated configurations of BDQ during this simulation: cluster 1 (left): 92%; cluster 2 (right): 5%. Non-polar hydrogen atoms are omitted for clarity.

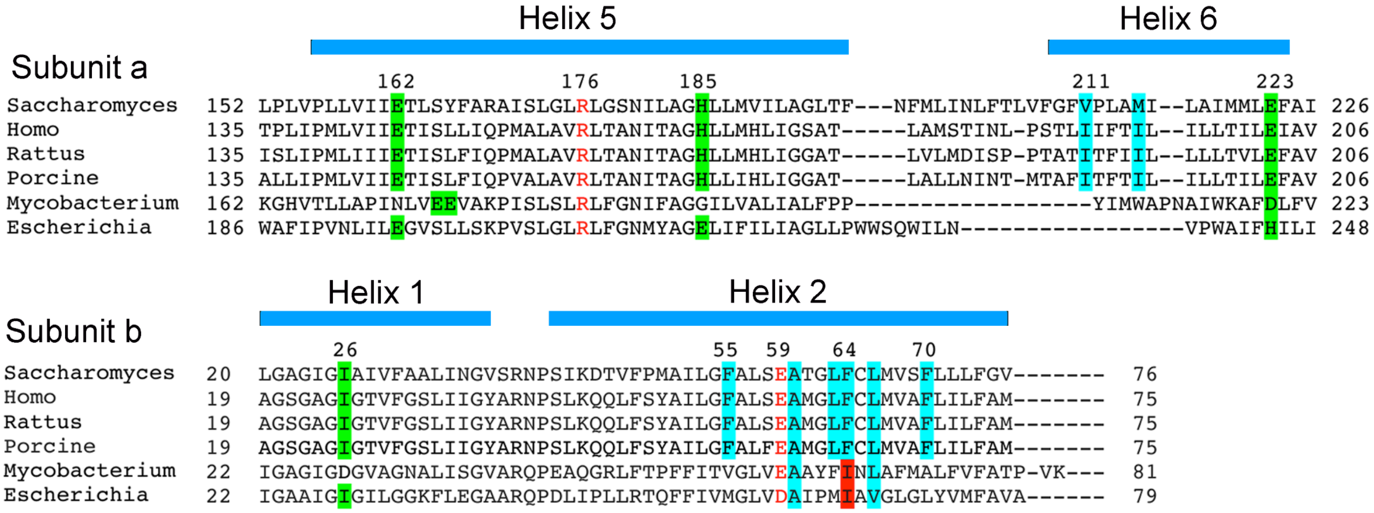

Supplement Figure 7. Primary sequence alignment within the putative bedaquiline binding site. The resides colored yellow/red are those that are in or around the density attributed to BDQ within yeast F_1F_o. Those residues in purple diverge in from those in the binding site, those colored green are important in the proton pathway. The area shaded is variable loop in subunit a that might affect the binding affinity of BDQ to site**.**
