# Supplementary material for: Bedaquiline inhibits the yeast and human mitochondrial ATP synthases
Source: Commun Biol. 2020 Aug 19;3:452. doi: 10.1038/s42003-020-01173-z (PMC7438494; doi:10.1038/s42003-020-01173-z)
Supplement: Supplementary file 2 — Description of Additional Supplementary Files [file 42003_2020_1173_MOESM2_ESM.pdf]

Description of Additional Supplementary Files

File Name: **Supplementary Movie 1**
Description: CryoEM map shown at different contour levels, as indicated, with the EM density attributed to BDQ in purple, c10-ring in yellow, subunit-a in orange, and the nanodisc in grey.

File Name: **Supplementary Data 1**
Description: contains the source data underlying thegraphs and charts presented in the main figures for the biochemistry in an Excel file
